# IrSPI, a Tick Serine Protease Inhibitor Involved in Tick Feeding and *Bartonella henselae* Infection

**DOI:** 10.1371/journal.pntd.0002993

**Published:** 2014-07-24

**Authors:** Xiang Ye Liu, Jose de la Fuente, Martine Cote, Ruth C. Galindo, Sara Moutailler, Muriel Vayssier-Taussat, Sarah I. Bonnet

**Affiliations:** 1 USC INRA Bartonella-Tiques, French National Institute of Agricultural Research (UMR BIPAR ENVA-ANSES-UPEC), Maisons-Alfort, France; 2 SaBio. Instituto de Investigación en Recursos Cinegéticos IREC-CSIC-UCLM-JCCM, Ciudad Real, Spain; 3 Department of Veterinary Pathobiology, Center for Veterinary Health Sciences, Oklahoma State University, Stillwater, Oklahoma, United States of America; University of California San Diego School of Medicine, United States of America

## Abstract

*Ixodes ricinus* is the most widespread and abundant tick in Europe, frequently bites humans, and is the vector of several pathogens including those responsible for Lyme disease, Tick-Borne Encephalitis, anaplasmosis, babesiosis and bartonellosis. These tick-borne pathogens are transmitted to vertebrate hosts via tick saliva during blood feeding, and tick salivary gland (SG) factors are likely implicated in transmission. In order to identify such tick factors, we characterized the transcriptome of female *I. ricinus* SGs using next generation sequencing techniques, and compared transcriptomes between *Bartonella henselae*-infected and non-infected ticks. High-throughput sequencing of *I. ricinus* SG transcriptomes led to the generation of 24,539 isotigs. Among them, 829 and 517 transcripts were either significantly up- or down-regulated respectively, in response to bacterial infection. Searches based on sequence identity showed that among the differentially expressed transcripts, 161 transcripts corresponded to nine groups of previously annotated tick SG gene families, while the others corresponded to genes of unknown function. Expression patterns of five selected genes belonging to the BPTI/Kunitz family of serine protease inhibitors, the tick salivary peptide group 1 protein, the salp15 super-family, and the arthropod defensin family, were validated by qRT-PCR. *IrSPI*, a member of the BPTI/Kunitz family of serine protease inhibitors, showed the highest up-regulation in SGs in response to *Bartonella* infection. *IrSPI* silencing impaired tick feeding, as well as resulted in reduced bacterial load in tick SGs. This study provides a comprehensive analysis of *I. ricinus* SG transcriptome and contributes significant genomic information about this important disease vector. This in-depth knowledge will enable a better understanding of the molecular interactions between ticks and tick-borne pathogens, and identifies IrSPI, a candidate to study now in detail to estimate its potentialities as vaccine against the ticks and the pathogens they transmit.

## Author Summary

*I. ricinus* is the most common tick species in Europe and the vector for several pathogens, including bacteria from the *Bartonella* genus. The mechanisms by which ticks modulate their gene expression in response to pathogen infection are poorly understood. In this report, we compared the differential expression of genes expressed in tick salivary glands during *B. henselae* infection using next generation sequencing techniques. This approach identified 829 and 517 transcripts either significantly up- or down-regulated respectively, in response to bacterial infection. Among them, 161 transcripts corresponded to nine gene family groups previously described in ticks. By silencing the most up-regulated transcript (*IrSPI*), we demonstrated its implication in both tick feeding and bacterial infection of tick salivary glands. This study demonstrates that a molecular dialogue exists between pathogens and their arthropod vectors and provides, with *IrSPI*, a candidate to study now in detail to estimate its potentialities as vaccine against the ticks and the pathogens they transmit.

## Introduction

Tick-borne diseases represent an increasing threat to human and animal health. Ticks are obligate blood-feeding ectoparasites of vertebrates, and can transmit pathogens such as viruses, bacteria and protozoa to both humans and animals. The hard tick *Ixodes ricinus* (Acari: *Ixodidae*) is one of the most common tick species in Western Europe. It is frequently associated with bites in humans, and is, among other tick species, a vector for the Tick-Borne Encephalitis virus, *Babesia* spp., *Borrelia burgdorferi s.l.*, *Rickettsia* spp., and *Anaplasma phagocytophilum* [1]. Whether ticks are involved in the transmission of *Bartonella* spp. has been heartily debated for many years due to plentiful but only indirect evidence of potential transmission (see reviews by [2]–[4]). We have demonstrated that *I. ricinus* is a competent vector for both *Bartonella henselae ex vivo* and for *Bartonella birtlesii in vivo*, and both systems constitute a good model for studying the modalities of pathogen transmission by ticks [5], [6]. *Bartonella* species are facultative intracellular gram-negative bacteria that are responsible for several diseases in humans and animals, and are becoming more frequently linked with several symptoms, particularly ocular infections and endocarditis (see review in [7]).

Current tick control strategies essentially rely on the use of chemical acaricides and repellents, therefore new approaches that are environmentally safe and that can provide broad protection against both current and novel tick-borne pathogens are urgently needed. One attractive solution is to develop vaccine strategies that target conserved tick components playing key roles in vector infestation or vector capacity [8]. Tick factors can effectively elicit protective immune responses when used to vaccinate against tick-borne diseases, as demonstrated in recent studies [9], [10].

Compared with other haematophagous arthropods, feeding in ixodid ticks is a slow and complex process, taking several days until tick repletion and detachment from the host [11]. This prolonged period of host attachment has sparked great interest in studying tick salivary gland (SG) secretions during feeding. As with other haematophagous arthropods, ticks face host hemostasis, inflammation and adaptive immunity during the blood-feeding process, and have consequently evolved a complex and sophisticated pharmacological armament against these potentially harmful processes. Accordingly, tick saliva contains anti-clotting, anti-platelet aggregation, vasodilator, anti-inflammatory and immunomodulatory components that allow ticks to successfully feed (see review in [12]). Tick-borne pathogens are injected into the vertebrate host concurrently with tick saliva during the blood meal, and salivary components favor pathogen transmission by interfering with host immunological responses [13]. In addition, several studies report that tick SGs differentially express transcripts in response to pathogen infection, some of which correspond to proteins implicated in pathogen transmission (see review in [14]). As the primary rate-limiting step in the development of anti-tick vaccines is the identification of protective antigenic targets [15], analyzing whole tick SG transcriptomes offers a straightforward approach to discovering such antigens.

Among ticks of medical and veterinary interest, many SG transcriptome analyses have been performed using traditional Sanger sequencing methods (review in [14]). More recently, next generation sequencing (NGS) techniques permit higher transcriptome coverage and more in-depth information on rare tick transcripts. Indeed, using NGS, Schwarz A, *et al.* described 272,220 contigs arising from SG transcriptomes of early- and late-feeding *I. ricinus* nymphs or adults [16].

The aim of this study was to analyze tick SG transcriptomes using NGS to identify proteins potentially involved in either bacterial development or transmission to the vertebrate host. Such information will improve our understanding of the molecular interaction between ticks and tick-borne pathogens, and will help identify potential vaccine candidates for the control of tick-borne diseases. We chose the combination of *B. henselae* - *Ixodes ricinus* as a model of controlled pathogen infection in this tick species. We hypothesized that if bacterial infection regulates the expression of specific tick SG genes, then these genes are likely to be implicated in the transmission of said bacteria. To address this proposition, we compared SG transcriptomes from infected and non-infected ticks. Sequences of differentially expressed genes were analyzed and compared to genes known to be implicated in tick-borne pathogen transmission in other models. Among up-regulated transcripts, we showed that silencing *IrSPI*, encoding a serine protease inhibitor, correlates with decreased *B. henselae* SG infection, and also alters tick feeding. These data suggest that *IrSPI* has a potential as an effective vaccine candidate to control tick burden and tick-borne disease, and that this candidate deserves a deeper interest in the future to estimate such potentialities.

## Materials and Methods

### Ticks and bacterial strains

All pathogen-free *I. ricinus* larvae were derived from a laboratory colony reared at 22°C and in 95% relative humidity with 12 h light/dark cycles as previously described [5]. *B. henselae* (Houston-1 ATTCC 49882) was grown in 5% defibrinated sheep blood Columbia agar (CBA) plates incubated at 35°C in an atmosphere of 5% CO_2_. After 7 days, bacteria were harvested and suspended in sterile phosphate-buffered saline (PBS) before being used for artificial tick feeding [5].

### Tick sample preparation

The method of artificial feeding used in this study was previously described [17]. Briefly, 5 mL of sheep blood (BioMèrieux, Lyon, France) were added into feeders and changed twice daily until tick repletion. *B. henselae*-infected sheep blood was prepared by adding 5 µL of the *B. henselae* suspension at a concentration of 10^9^ CFU/mL in PBS, to 5 mL sheep blood, resulting in a final bacterial concentration similar to that found in naturally infected hosts. The same protocol was then applied in order to engorge *B. henselae*-infected nymphs, born from infected larvae. Nymphs were then allowed to molt into adult females or males. Following this protocol of feeding larval and nymph stages on *B. henselae-*infected blood, all the resulting adults became infected with Bartonella. To induce multiplication and/or migration of *B. henselae* inside SGs [5], the resulting Bartonella-infected *I. ricinus* (BIr) females were fed for 4 days with bacteria-free blood before being dissected. Production of non-infected *I. ricinus* (NIr) followed the same blood feeding procedure using non-infected blood at all stages. SGs were dissected on ice under a stereomicroscope in sterile ice-cold 1× PBS, briefly rinsed and then immediately stored at −80°C until total RNA extraction and sequencing. The same protocol was used to produce ticks used in qRT-PCR and RNAi experiments.

### Total RNA extraction

Total SG RNA was isolated using TRIzol Reagent (Invitrogen, USA), RQ1 RNase-free DNase (Promega, USA) and RNasin Ribonuclease Inhibitor (Promega, USA) following the manufacturer's instructions. Quality and quantity of total RNA was assessed on a Nanodrop 2000 machine (Thermo SCIENTIFIC, USA). For each experimental condition (BIr and NIr), 30 µg total RNA was obtained per sample, isolated from 69 pairs of salivary glands, and used for transcriptomic sequencing. RNA samples used in qRT-PCR experiments were prepared in the same manner.

### High throughput sequencing of *B. henselae*-infected and non-infected *I. ricinus* salivary gland transcripts

To generate an *I. ricinus* SG reference transcriptome, total RNA from BIr and NIr SGs were pooled at equimolar concentrations and cDNA libraries were constructed and normalized prior to sequencing on the GS FLX Titanium platform (454 pyrosequencing, Roche, CT, USA) by GATC Biotech AG (Konstanz, Germany). After trimming sequencing primers and adapter, *de novo* assembly of all reads was performed with GS *De Novo* Assembler Software version V2.5.3 (454 Life Science Corp, CT, USA), and assembly results were reported as contigs and isotigs. A contig represent a set of overlapping DNA segments that together represent a consensus region of DNA. An isotig is meant to be analogous to an individual transcript. Different isotigs from a given isogroup (group assembly of different transcripts) can be inferred splice-variants. The reported isotigs are the putative transcripts that can be constructed using overlapping reads provided as input to the assembler.

To compare transcriptomes from BIr-SGs and NIr-SGs, samples were first treated with ultrasound (4 cycles at 4°C for 30 s) and resulting fragmented RNA samples were then treated with polynucleotide kinase. The poly (A)-tailed 3′-RNA fragments from each total RNA sample were then isolated using oligo-dT chromatography, and first-strand cDNA synthesis was performed using an RNA adaptator and an oligo-dT primer. The 250–450 bp 3′UTR cDNA libraries were then separately sequenced on the HiSeq2000 by GATC Biotech AG. Reads (50 bp) per sample from all runs were concatenated and polyA tails were trimmed.

All the obtained sequences were submitted to GenBank and registered with the BioProject database (Short Read Archives (SRA), BioProject ID PRJNA237360).

### Transcript annotation

All isotigs were imported into the BLAST2GO version 2.5.0 (www.blast2go.org) program for searches based on sequence identity and Gene Ontology (GO) annotation. In the identity searches, isotigs were compared against the NCBI nr protein database using BlastX with an E-value cutoff of 1.0E-10. The BLAST results were used to map the consensus sequences with GO terms and to summarize the distribution of the sequences into three main categories: Biological Processes (BP), Cellular Components (CC) and Molecular Functions (MF).

The KEGG (Kyoto Encyclopedia of Genes and Genomes) automatic annotation server was used for gene ortholog assignment and pathway mapping for all isotigs. Depending on the similarity of hits against the KEGG database using BlastX, the isotigs were then assigned unique enzyme commission (EC) numbers. Distribution of isotigs under the respective EC numbers was then used to map them to the KEGG biochemical pathway.

### Analysis of differentially expressed transcripts between *B. henselae*-infected and non-infected *I. ricinus* salivary glands

Burrows-Wheeler Transform Aligner (BWA) [18] was used to align polyA trimmed HiSeq2000 reads against the *I. ricinus* SG reference transcriptome, i.e. the isotig data produced by 454 pyrosequencing. The resulting sequence alignment/map was used to calculate counts (number of reads that mapped to the reference).

Counts per isotig were assessed in both BIr-SG and NIr-SG samples. Isotigs with fewer than five counts were eliminated. To calculate the relative expression profiles in infected ticks, relative abundance (RA) values were computed for each isotig per sample by dividing its sequence count by the total sequence count for each sample. Differentially expressed isotigs between infected and non-infected ticks were detected with R [19] and χ^2^ statistics tests with Bonferroni correction using IDEG6 software (http://telethon.bio.unipd.it/bioinfo/IDEG6_form/) [20]. An isotig was considered to be significantly differentially expressed in response to *B. henselae* infection when the RA had a fold change (FC) ≥2.0 and both statistical tests yielded significant values at P≤0.0001.

The open reading frame (ORF) of differentially expressed isotigs was determined using the ORF finder webserver at www.ncbi.nlm.nih.gov/projects/gorf. Conserved domains of each differentially expressed isotig were found using the conserved domains database (CDD) webserver version v3.03 at www.ncbi.nlm.nih.gov/Structure/cdd/cdd.shtml.

### Real time quantitative PCR

Validation of the expression profiles of selected genes was performed by real time quantitative RT-PCR (qRT-PCR) on SG RNA samples prepared similarly to those used for the differential transcriptome analysis, as biological replicates. First-strand cDNA was synthesized with the SuperScript III First-Strand Synthesis system for RT-PCR kit (Invitrogen) from 400 ng total RNA. Each qPCR reaction was performed in a total volume of 12 µL with 0.2× LightCycler 480 DNA SYBR Green I Master Mix (Roche), 10 µM of each primer and 2 µL of template. Reactions were run using the Roche LightCycler 480 System under the following conditions: 95°C 5 min; 95°C, 10 s, 60°C 15 s, 72°C 15 s, 45cycles. Each sample was run in triplicate with results analyzed by Roche LightCycler 480 Software V1.5.0. Relative quantification of gene expression was calculated using the comparative Ct method [21]. The results were then normalized using the *I. ricinus* actin gene, and sequence-specific primers used for qPCR are listed in Table 1. Statistical analysis was performed using two-tailed Student's *t* tests and values were considered significant at *p*≤0.0001. Data analysis was performed with Prism 5.0 (GraphPad Software, Inc. USA), and results were expressed as mean ± SEM (standard error of the mean).

**Table 1:** List of qPCR primers and siRNA sequences used in this article, and the accession number of the corresponding *I. ricinus* gene.

Accession No.	Description	Used/Expected expression	Sense/anti-sense (5′-3′) primers
**KF531922**	IrSPI BPTI/Kunitz family of serine protease inhibitor	qPCR/Up-regulated	TCTTCGCTGCTGTCTCGTAC CCTTCAAAGGCTCGCATTGG
**KF531923**	Tick salivary peptide group 1 protein (20 kDa)	qPCR/Down-regulated	CAGCGACATTTCTCGGTGTAT CCATTTCCAGTTGTGCAATCG
**KF531924**	Salp15 super-family protein	qPCR/Up-regulated	CAAGACTGATCGTGGCAATGT CTTTTAGCGCACCAAGGGTAT
**KF531925**	Salp15 super-family protein	qPCR/Down-regulated	GAACTCGTGGACATTTGCCAA GTTTCGGGGCATCTCTAGTG
**KF531926**	Arthropod defensin	qPCR/Down-regulated	TGAAAATGACGAGGGAGGAGA TGAACAAGATGCAGGTCCTTT
**AJ889837.1**	*I. ricinus* actin	qPCR internal control	ACGGGTATCGTGCTCGACT TCAGGTAGTCGGTCAGGTC
**AF369529.1**	*B. henselae* ITS	qPCR	AGATGATGATCCCAAGCCTTCTGG GATAAACCGGAAAACCTTCCC
**KF531922**	IrSPI	siRNA/Up-regulated	GCUAAACUUAGAACUGUCUACUCCU AGGAGUAGACAGUUCUAAGUUUAGC

### *IrSPI* silencing by RNA interference

siRNAs targeting *IrSPI* were designed using the E-RNAi Web service (www.dkfz.de/signaling/e-rnai3/idseq.php) (Table 1) and were synthesized *in vitro* using the Stealth RNAi siRNA construction kit (Life technologies, France). Four nL (25 µM) of siRNA (∼10^13^ molecules) were injected into the tick hemoceole of *B. henselae*-infected females (infected both at larval and nymph stages) prior to feeding on an uninfected blood meal for 7 days. Injections were performed as previously described [22]. Control ticks received 4 nL of nuclease-free water (Life technologies, France). Silencing was verified on SG RNA extracted from mock-injected and experimental ticks (see below).

Two phenotypes were analyzed after *IrSPI* siRNA injections: 1) feeding rates and 2) *B. henselae* loads in SG. To estimate feeding rates, eight ticks per group (mock and siRNA injections) were used. After seven days of feeding, ticks were weighed and results compared between siRNA-injected groups and controls, by Student's *t* test with unequal variance. Ticks were then dissected to quantify both *IrSPI* silencing and *B. henselae* loads in SGs. For each pair of SGs, one gland was used for gene silencing quantification by qRT-PCR and the other gland for *B. henselae* quantification by DNA qPCR. RNA was extracted as described previously, and DNA was extracted using the Wizard genomic DNA purification kit (Promega, USA) following manufacturer's instructions. *B. henselae* was quantified using primers targeting the 16S–23S intergenic spacer (ITS) [23] (Table 1). Quantitative PCR results were assessed by extrapolation from the standard curve and normalized to *I. ricinus* actin. Statistical analysis of qPCR was performed using two-tailed Student's *t* tests. A *p* value<0.05 was scored as a significant difference. Data analysis was performed with Prism 5.0 (GraphPad Software, Inc. USA), qPCRs were performed in triplicate and results expressed as mean ± SEM.

## Results

### Production of *B. henselae*-infected ticks

The engorgement of 4,548 larvae and resulting nymphs with *B. henselae*-infected or non-infected sheep blood resulted in a total of 110 *B. henselae*-infected *I. ricinus* females and 109 non-infected *I. ricinus* females. Of these, 69 from each group were used for SG preparation and total RNA isolation. An average of 590 ng total RNA per SG pair was obtained.

### *I. ricinus* salivary gland transcriptome analysis

**Figure 1:**
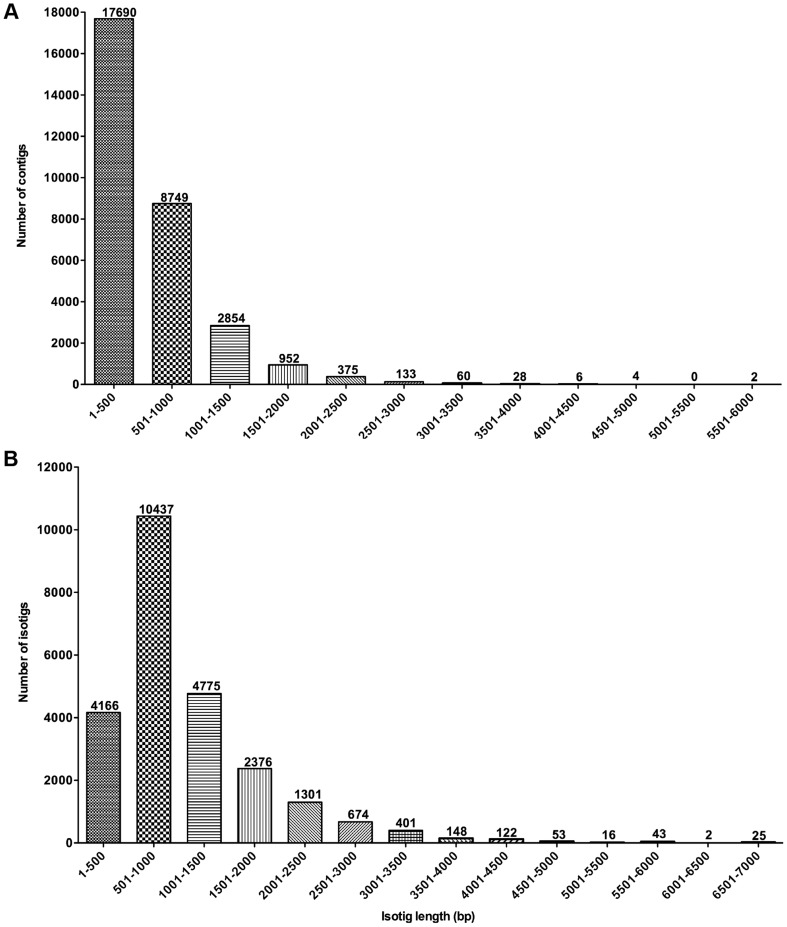
Size description of transcripts generated by *de novo* assembly of the quality filtered and trimmed 454 pyrosequencing reads using GS *de novo* assembler version 2.5.3 from *B. henselae*-infected and non-infected *I. ricinus* female salivary glands: A) contigs, B) isotigs.

To obtain a high coverage of tick SG transcripts, normalized cDNA libraries from BIr-SGs and NIr-SGs were sequenced twice using the GS FLX titanium platform. After trimming and removing superfluous sequences (primers and adapters), all reads were used for transcript assembly, generating 30,853 contigs and 15,756 isogroups composed of 24,539 isotigs (Table 2). The description of contig and isotig sizes are shown in Figures 1A and 1B respectively.

**Table 2:** Summary and *de novo* assembly of *I. ricinus* salivary gland transcriptome sequenced by 454 Pyrosequencing.

	*Sequences*	*Largest length (bp)*	*Average length (bp)*	*N50 (bp)*	*GC%*	*Total bases*
**Sequenced**	778,598	1,185	379	436	45.05	295,181,527
**Trimmed**	780,228	1,164	353	411	44.46	276,127,075
**Contigs**	30,853	5,647	550	1,026		16,970,400
**Isotigs**	24,539	6,815	1,100	1,348		26,884,585

**Figure 2:**
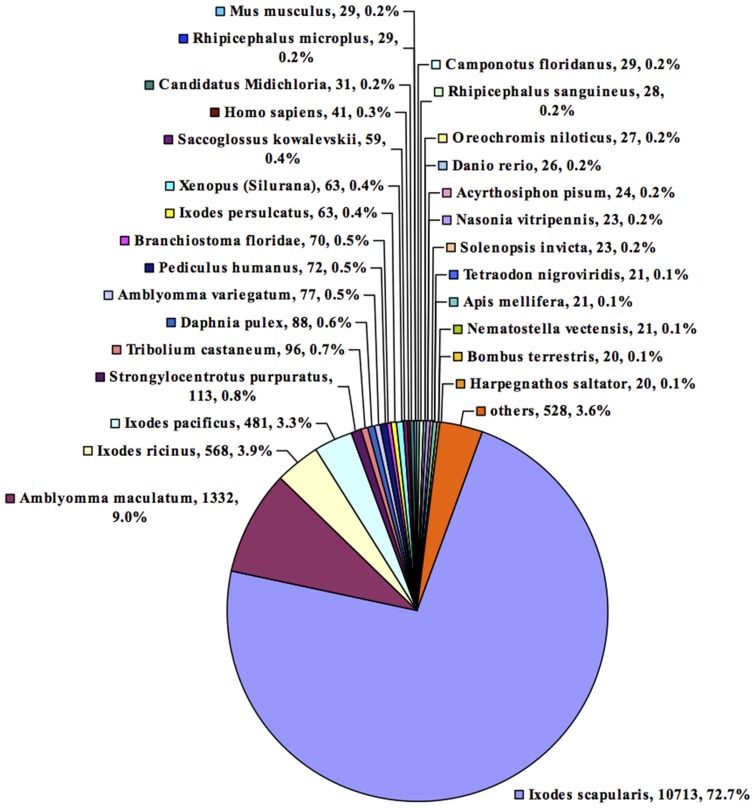
Percentage similarity distribution of transcripts expressed in *B. henselae*-infected and non-infected *I. ricinus* female salivary glands, from the top hit in the protein database.

Sequence identity percentages between translated *I. ricinus* SG isotigs and the nr protein database were identified with BlastX using Blast2GO software. Out of the 24,539 assembled isotig sequences, 14,736 sequences (60.1%) had significant similarity (E-value≤1E-10) with sequences present in GenBank. Of these, 10,713 (72.7%) had closest alignment with *Ixodes scapularis* sequences, 1,332 (9.0%) with *Amblyomma maculatum* sequences, 568 (3.9%) with *I. ricinus*, 481 (3.3%) with *Ixodes pacificus* sequences, and 63 (0.4%) with *Ixodes persulcatus* sequences (Figure 2).

**Figure 3:**
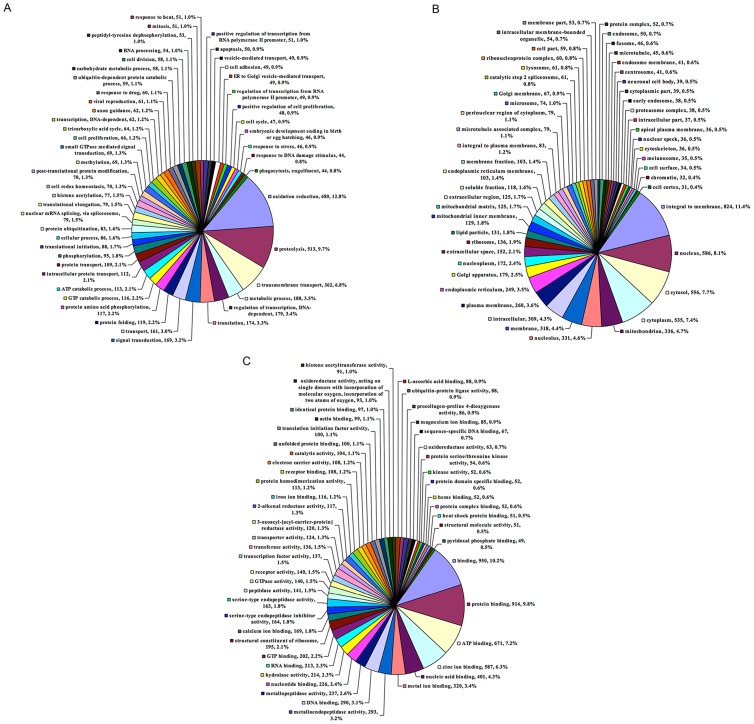
Gene ontology assignments of transcripts expressed in *B. henselae-*infected and non-infected *I. ricinus* female salivary glands: A) Biological progresses, B) Cellular components, C) Molecular functions.

The database of Blast results was then used to annotate the isotigs with GO terms. Isotigs were classified according to the categories of biological processes (BP) in which they may be implicated, the cellular components (CC) with which they may be linked, or a related molecular function (MF). One or more GO IDs were assigned to 10,859 (44.3%) isotigs. The number of isotigs that could be annotated as belonging to either BP, CC and MF categories were 5,308, 7,213 and 9,283, respectively. In the BP category, ‘oxidation reduction’ (12.8%) was the most abundant GO term, followed by ‘proteolysis’ (9.7%) (Figure 3A). In the CC category, the most abundant term was ‘integral to membrane’ (11.4%), followed by ‘nucleus’ (8.1%), ‘cytosol’ (7.7%) and ‘cytoplasm localization’ (7.4%) (Figure 3B). In the MF category, the most abundant term was ‘binding proteins’ (63.2%) (Figure 3C).

Isotigs were allocated to various biological pathways using the KEGG server, and several isotigs were assigned more than one pathway. Out of the 24,539 isotigs analyzed, 2,465 may be implicated in metabolic pathways such as C5-branched dibasic acid, ether lipid, starch and sucrose, or fatty acid metabolism. An additional 936 mapped isotigs were thought to be implicated in biosynthetic pathways for such molecules as cutin, suberine, wax, glycosylphosphatidylinositol (GPI)-anchor, novobiocin, phenylalanine, tyrosine and tryptophan. Finally, 1,095 mapped isotigs may be linked to 33 other pathways such as glycolysis/gluconeogenesis, benzoate degradation, and synthesis and degradation of ketone bodies.

### Analysis of differently expressed transcripts between *B. henselae*-infected and non-infected tick salivary glands

In order to investigate the differential expression of transcripts between BIr-SGs and NIr-SGs, corresponding 3′UTR cDNA libraries were sequenced, which generated 210 and 150 million raw sequences reads, respectively. Isotigs with an RA fold change (FC) of ≥2 and χ^2^≤0.0001 were selected as significantly differentially expressed, resulting in 5.5% of isotigs (1,346/24,539) which varied in their expression following *B. henselae* infection (Additional file 1). Of these, 829 isotigs were up-regulated in *B. henselae*-infected ticks and 517 isotigs were down-regulated after bacterial infection.

Based on translated sequence identity with protein database sequences, isotigs were classified into three groups: (a) proteins with identity to proteins of known function, (b) proteins with identity to proteins of unknown function and (c) proteins without identity (Additional file 1 and Table 3). Among the first group, proteins were classified into nine protein families, out of which four contained both up-regulated and down-regulated transcripts in response to pathogen infection, while five corresponded to transcripts that were only down-regulated in response to infection (Table 3).

**Table 3:** Differentially expressed families of transcripts in *B. henselae-*infected *I. ricinus* female salivary glands compared to non-infected salivary glands.

*Hypothetical proteins*	*Number of isotigs*	
	*Up-regulated*	*Down-regulated*
**Proteins of known function**		
Anti-complement proteins	0	10
Arthropod defensins	0	4
BPTI/Kunitz family of serine protease inhibitors	7	17
Collagen-Like Salivary secreted Peptide (CLSP)	0	6
Heat Shock Proteins (HSP)	0	9
Microplusin proteins	0	6
Salp15 super-family proteins	24	32
Tick Histamine Binding Proteins (THBPs)	14	26
Zinc-dependent metalloproteases	2	4
**Proteins of unknown function**		
A*mblyomma maculatum* Hypothetical proteins	27	10
*Daphnia pulex* Hypothetical proteins	4	0
*Ixodes scapularis* Hypothetical proteins	93	19
Ixodid Secreted salivary gland proteins	55	156
*Ixodes scapularis* Conserved hypothetical proteins	34	3
Ixodid proteins	164	60
Other species proteins	32	9
Tick Salivary Peptide Group 1 (TSPG-1)	12	30
Zinc finger proteins	14	0
**Unknown genes**	347	116
**Total**	**829**	**517**

**Figure 4:**
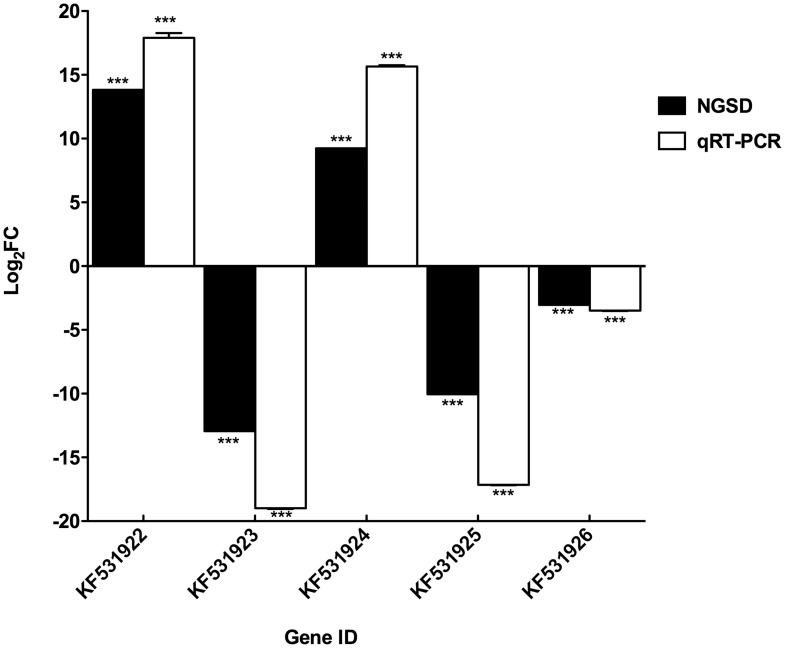
Comparison of the expression profile of five *I. ricinus* genes by next generation sequencing data (NGSD) and qRT-PCR analysis in *B. henselae*-infected and non-infected ticks. The figure shows differential expression of five genes. KF531922 (*IrSPI*) and KF531924 respectively associated with BPTI/Kuntiz family of serine protease inhibitors (*IrSPI*) and Salp15 superfamily protein, which were up-regulated in *B. henselae*-infected *I. ricinus* female SGs. KF531923, KF531925, and KF531926 respectively associated with tick salivary peptide group1 protein (20 kDa), Salp15 super-family protein, and arthropod defensins, which were down-regulated in *B. henselae*-infected *I. ricinus* female SGs. The fold changes (FC) were converted into log_2_ values. For qRT-PCR, each sample was run in triplicate and error bars show the SEM (standard error of the mean). The statistical tests yielded significant values at *** P≤0.0001.

The expression of five selected transcripts was validated by qRT-PCR on SG RNA samples from a biological replicate (Figure 4). Two transcripts corresponding to the BPTI/Kuntiz family of serine protease inhibitors (GenBank accession number: KF531922) and Salp15 superfamily protein (GenBank accession number: KF531924), respectively were overexpressed by *B. henselae* infection; three transcripts, corresponding to tick salivary peptide group1 protein (GenBank accession number: KF531923), Salp15 superfamily protein (GenBank accession number: KF531925), and arthropod defensins (GenBank accession number: KF531926), respectively, were down-regulated by *B. henselae* infection. The fold change (FC) calculation and statistical analysis (*p*≤0.0001) indicate a clear correlation between the transcript expression profile revealed by next generation sequencing-based data and transcript abundance analyzed by qRT-PCR (Figure 4).

### Silencing the *IrSPI* gene decreases tick feeding capacity as well as infection of tick SGs by *B. henselae*

**Figure 5:**
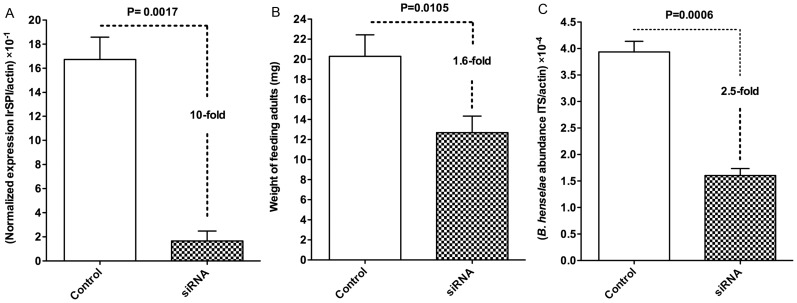
Influence of *IrSPI* silencing on tick feeding and tick SG infection by *B. henselae*. *IrSPI*-siRNA (siRNA) or nuclease free water (control) were microinjected into the body of *B. henselae*-infected *I. ricinus* females before ticks were fed a non-infected blood meal during 7 days. A) Quantitative RT-PCR analysis of *IrSPI* gene expression levels in pools of eight tick SGs from *IrSPI*-siRNA injected ticks and controls. The results are represented as the mean ± SEM of qRT-PCR performed in triplicate. B) Weight evaluation of *IrSPI*-siRNA injected tick body mass compared to controls. The results are represented as the mean ± SEM of eight ticks weighed individually. C) Quantitative PCR analysis of bacterial load in pools of eight tick SGs from *IrSPI*-siRNA injected ticks and controls. The results are represented as the mean ± SEM of qPCR performed in triplicate.

The transcript encoding a BPTI/Kunitz type serine protease inhibitor, thereafter named *IrSPI* (for *I. ricinus* serine protease inhibitor; GenBank accession number: KF531922), that was overexpressed in response to bacteril infection was selected for functional analysis in ticks. RNAi was used to evaluate the effect of *IrSPI* silencing on tick feeding as well as tick salivary gland infection by *B. henselae*. siRNA injection was performed in females arising from infected nymphs and larvae, prior to the provision of an additional uninfected blood meal, to ensure “total” SG infection of the adult females (see material and methods section). *IrSPI* transcript abundance was suppressed by 90% (*p* = 0.001) in ticks that received *IrSPI*-siRNA compared to ticks that only received control injections (Figure 5A). The mean weight of siRNA-injected females was significantly decreased by 1.6-fold (12.7 mg±1.7 vs. 20.3 mg±2.1), when compared to controls (Figure 5B). *B. henselae* load within SGs was significantly reduced by 2.5-fold in *IrSPI*-siRNA-injected ticks when compared to mock injected ticks (1.6×10^−4^±0.1 and 3.9×10^−4^±0.2 per actin gene copy, respectively) (Figure 5C).

## Discussion

Recent NGS advances enabled us to perform an exhaustive analysis of the *I. ricinus* SG transcriptome following infection with *B. henselae*, as a relevant model system for studying tick-pathogen interactions.

This analysis highlights the existence of major groups of genes which vary in response to infection, including those encoding proteins involved in protein binding, oxidation reduction or proteolysis, or that are integral to membranes, either nuclear or cytoplasmic. These results provide a reference databank for the *I. ricinus* SG transcriptome, which is particularly relevant in the absence of an *I. ricinus* genome sequence. Equally importantly this study provides abundant genetic information on the *I. ricinus* response to pathogen infection. Until now, most studies of tick SG transcriptomes were based on EST analyses [14]. Recently, using NGS, Schwarz *et al.* [16] investigated *I. ricinus* SG transcript variation during the transition from early- to late-feeding nymphs or adults. The study provided a higher transcriptome coverage than classical methodologies and increased the available genomic information for *I. ricinus*. However, the fact that, in this study, half the contigs are less than 200 bp may suggest potential problems of assembly. In addition in their study, ticks were collected in the field, had fed on various animals and there was no indication of the infection status of the vertebrate hosts from which the ticks were collected. Therefore, the reported transcriptome dynamics during attachment to the host should be considered with caution in the absence of tick infection status.

In the present report, we found that 5.5% of the identified isotigs varied in their expression level during *B. henselae* infection, reflecting probable molecular interactions between the pathogen and the vector. Among these isotigs, 62% were up-regulated, whereas 38% were down-regulated during bacterial infection. The balance observed between up- or down-regulated genes suggests a co-evolutionary mechanism, which could guarantee both pathogen transmission and vector survival. Database searches for sequence identity led to the classification of translated sequences into nine families, however it is important to keep in mind that belonging to the same family does not necessarily imply having the same function. Conversely, changes in transcript expression profiles do not necessarily translate into variation in the corresponding proteins expression.

## 

### BPTI/Kunitz family

Seven up-regulated and seventeen down-regulated isotigs in response to *B. henselae* infection contain BPTI/Kunitz domains (Acc CDD: cl00101). GO molecular function analysis showed that all isotigs of this group except one (isotig20663, which showed extracellular matrix structural constituent function) have serine-type endopeptidase or peptidase inhibitor signatures. No biological pathways were identified for this group of isotigs. With phylogenetic analysis, Schwarz *et al.* [13] recently demonstrated that proteins with more than three Kunitz domains appeared widely distributed across different tick species and, amongst all arthropods, have evolved only in ticks. In hard ticks, BPTI/Kunitz proteins can modulate blood feeding, disrupt host angiogenesis and wound healing [24]. These proteins are considered vital for hard tick survival, and constitute a potential therapeutic target against ticks and tick-borne pathogen transmission [24]. According to Schwarz *et al.* [13], they belong to the class of protease inhibitors that are the most highly secreted group of proteins in the *I. ricinus* SG transcriptome. Using the cysteine patterns of BPTI/Kunitz, Dai *et al.* [25] clustered 80 ixodid tick BPTI/Kunitz proteins into three clades (groups I, II and III). In *I. scapularis* and *I. ricinus*, genes from group II are expressed in the middle and late stages of blood feeding, with the exception of the *Isc.218* gene which begins to be expressed at 6–12 hours, increases strikingly at 18–24 hours and then decreases rapidly at 72 hours after tick attachment, while genes from group III are only expressed in the late stages of blood feeding [25]. The expression of BPTI/Kunitz proteins is thus a dynamic process during long-term blood feeding, a fact that may contribute to the finding of both up- and down-regulated BPTI/Kunitz serine protease inhibitor genes during *B. henselae* infection.

We found that *IrSPI* encodes for a protein of the BPTI/Kunitz family and exhibited the highest differential up-regulation in *Bartonella*-infected SG. A similar up-regulation was reported for a homologous protein: *DvKPI* (*Dermacentor variabilis* kunitz protease inhibitor) in *D. variabilis* tick midgut infected with *R. montanensis* [26]. *IrSPI* silencing experiments confirmed that this protein likely contributes to tick blood feeding as tick weight decreased when expression of *IrSPI* was impaired. Furthermore, our results also showed that *IrSPI* has an impact on *B. henselae* development in *I. ricinus*, as silencing *IrSPI* decreases *B. henselae* loads in *I. ricinus* SGs. In contrast, silencing the *DvKPI* gene in *D. variabilis*, enhanced rickettsial colonization of the tick midgut, suggesting that this protein is implicated in the defense response limiting *R. montanensis* invasion [27]. These differences could be explained by the various regulations observed for proteins belonging to the BPTI/Kunitz family, perhaps reflecting different functions. Our current view is that *IrSPI* is putatively involved with *B. henselae* adhesion/invasion/multiplication, but might also be involved with the stress/defense response, similar to *DvKPI*. Indeed, its over-expression following infection with *B. henselae* may decrease the amount of other bacterial species in competition with *B. henselae*, facilitating its establishment in the SGs. A similar result has been reported for *D. variabilis*, as silencing expression of varisin, belonging to the defensin superfamily, reduced *A. marginale* infection [28]. These results highlight the role of IrSPI during tick feeding and *B. henselae* infection but further investigations are needed to elucidate the role of *IrSPI* and to evaluate the vaccine potential of this molecule in the context of an anti-tick and a transmission-blocking vaccine against *B. henselae* and other tick-borne pathogens.

### Anti-complement proteins family

In addition to isotigs encoding for proteins containing BPTI/Kunitz domains, other protein families were identified among differentially expressed isotigs. Ten isotigs which were down-regulated in response to *B. henselae* infection presented high similarity with sequences corresponding to IxAC (*Ixodes* anti-complement) proteins, that are implicated in tick blood feeding processes [29]. None of these showed any functional domains, GO terms or link to a biological pathway, but their high similarity with anti-complement proteins of ixodid ticks (82–100%) suggested anti-complement activity. Such activity has been already identified in *I. ricinus* SG extracts [29] and some anti-complement proteins have been shown to be up-regulated during blood feeding [30]. In our study, we found that those isotigs annotated as anti-complement proteins were down-regulated in response to *B. henselae* infection. One might wonder why the bacterium down-regulated anti-complement proteins, especially considering the harmful impact of the complement on *Bartonella* spp., unless *Bartonella* spp. uses its own defense system against the complement cascade [31].

### Arthropod defensing family

Four isotigs, all of which were down-regulated in response to *B. henselae* infection, harbored an arthropod defensin domain (Acc CDD: cl03093). Defensins are 3–4 kDa cationic antimicrobial peptides (AMPs) implicated in defense response processes [32], and are mainly expressed after blood feeding in the tick midgut, but also in other organs such as SGs and ovaries [33]. It has been reported that defensins are up-regulated in the midgut of *Ornithodoros moubata* after injection of *Escherichia coli* and *Micrococcus luteus* [34], [35]. In a similar manner, in *Dermacentor variabilis* ticks naturally infected with *Anaplasma marginale*, defensins are up-regulated after an injection of *E. coli*, *Bacillus subtilis* or *M. luteus* [36]. Interestingly, when ticks are infected with tick-borne pathogens, tick defensins display variable expression levels during blood feeding. In *Rickettsia montanensis*-infected *D. variabilis* ticks, one defensin transcript in the midgut was down-regulated at 18 hours post feeding, up-regulated between 24 and 48 hours, and then down-regulated again at 72 hours, whereas in the fat body, this transcript was down-regulated 48 hours prior to feeding, and up-regulated 72 hours after feeding [37]. It was also reported that one contig annotated as a defensin precursor was down-regulated in Langat virus (LGTV)-infected *I. scapularis* ticks [38]. Thus, it could be hypothesized that ticks up-regulate defensins as a protective response to infection with non-tick-borne pathogens. However, in the presence of tick-borne pathogens that have co-evolved with the tick vector, these pathogens are able to manipulate defensin expression in order to suppress tick immune responses, and prolong bacterial survival, multiplication and transmission, as reported here for *B. henselae*.

### Microplusin family

Six isotigs showed high (90–98%) similarity to the *I. scapularis* microplusin sequence, which belongs to an antimicrobial peptide gene family. All six isotigs were down-regulated in *B. henselae*-infected *I. ricinus* SGs. Functional domains, GO terms or biological pathways were not identified for these isotigs. Microplusins were first isolated from hemocytes, ovaries and fat body of the cattle tick *Rhipicephalus (Boophilus) microplus*, as antimicrobial peptides acting against the Gram-positive bacterium, *M. luteus*, and the yeast, *Cryptococcus neoformans* [39]. Recently, it was reported that two contigs annotated as Microplusin preprotein-like were down-regulated in Langat virus (LGTV)-infected *I. scapularis* ticks [38]. Finding isotigs with significant similarity to Microplusins, which were down-regulated after *Bartonella* infection, may suggest a possible co-evolutionary mechanism similar to that found with defensins.

### Collagen-like salivary secreted peptides family

Six isotigs that were down-regulated in response to *B. henselae* infection presented high similarity with sequences corresponding to *I. pacificus* collagen-like salivary secreted peptide (CLSP) (70–92%). Functional domains, GO terms or implication in specific biological pathways were not identified for these isotigs. As the CLSPs identified in *I. pacificus* are relatively glycine and proline rich, it was suggested that they could affect vascular biology and adhere to molecules that help with tick attachment to host skin [40]. However, the function and expression of CLSPs during blood feeding and pathogen transmission is unknown.

### Heat Shock proteins (HSP) family

Nine isotigs which were down-regulated in response to *B. henselae* infection showed some similarity with sequences coding for proteins involved in stress response biological processes. Among them, eight isotigs were highly similar to *I. scapularis* HSP20 protein (91–95%) and one to *I. scapularis* HSP70 protein (97%). Again, no connection to a potential biology pathway could be identified for any of the isotigs in this group. The heat shock response is a conserved reaction of both cells and organisms to high temperatures and other stress conditions, and is effected by HSPs [41]. It has been reported that HSP20 can protect tick cells from stress, impact tick behavior such as questing speed, and can be involved in the *I. scapularis* protective response to *A. phagocytophilum* infection [42], [43]. However, these studies also reported that in the natural vector-pathogen relationship, HSPs and other stress response proteins were not strongly activated, which is likely due to tick-pathogen co-evolution [43]. The complexity of the tick stress response to infection was also evidenced in the results reported here, suggesting that some pathogens may induce down-regulation of tick heat shock responses, likely increasing pathogen survival and multiplication.

### Salp15 super-family proteins

Twenty-four up-regulated and 32 down-regulated isotigs in response to *B. henselae* infection contained sequences similar to the salp15 super-family domain (Acc CDD: cl13541). No GO terms or biological pathways could be determined for any of them. The salp15 protein was first identified as an *I. scapularis* salivary protein with multiple functions, such as the inhibition of CD4^+^ T cell activation by specifically binding to the T cell co-receptor CD4 [44]–[46], as well as inhibition of cytokine expression by dendritic cells [47]. Salp15 has also been implicated in the protection of *Borrelia* species, the Lyme disease agent, from complement and antibody-mediated killing by the host, as well as allowing the bacteria to remain attached to tick cells [47], [48]. During *I. scapularis* blood feeding, it has been shown that salp15 mRNA and protein levels were 13-fold and 1.6-fold higher, respectively, in engorged tick SGs infected with *B. burgdorferi* [48]. In addition, RNA interference-mediated salp15 knockdown in *I. scapularis* drastically reduced the capacity of these ticks to transmit *Borrelia* spirochetes to mice [48]. These findings demonstrated that *Borrelia* sp. exploits the salp15 tick protein and is able to induce its expression in order to facilitate mammalian host infection. Based on these results reported for *Borrelia* [48], [49], it is then possible to speculate that *Bartonella* sp. are also capable of increasing the production of some of the salp15 proteins to facilitate their transmission to the vertebrate host.

### Tick histamine binding proteins family

Forty isotigs were also identified as harboring a tick histamine binding domain (Acc CDD: cl03446): 14 were up-regulated and 26 down-regulated in response to *B. henselae* infection. All showed a binding GO molecular function, but no implication in a cellular component or biological process/pathway could be identified. Histamine binding proteins (HBPs) are lipocalins with two binding sites. Lipocalins are small extracellular proteins that bind to histamine, serotonin and prostaglandin, and are implicated in the regulation of cell homeostasis and vertebrate immune responses [50], [51]. It has been reported that out of three closely related HBPs isolated from fed *R. appendiculatus* SGs, two—Ra-HBP1 and Ra-HBP2—are female specific, whereas Ra-HBP3 is exclusively secreted by larvae, nymphs and adult male ticks [52]. It has also been demonstrated that tick female-specific HBPs are found only during the early feeding period, peaking about 48 hours after tick infestation [52]. Such findings showed that HBP expression is a dynamic process during tick feeding and the results reported here with several up- and down-regulated genes after bacterial infection, suggest that HBPs might also be implicated in tick- *B. henselae* interactions.

### Zinc-dependent metalloproteases family

Two up-regulated and four down-regulated isotigs in response to *B. henselae* infection had a zinc-dependent metalloprotease domain (Acc CDD: cl00064). The two up-regulated isotigs (isotig09315 and isotig10110) showed hydrolase and peptidase signatures, respectively. All down-regulated isotigs shared the same metallopeptidase putative molecular function and two of them (isotig03163 and isotig07095) were implicated in proteolysis biological processes. No implication in a potential biological pathway could be identified for any other isotig in this group. Metalloproteases have been described in the SGs of *I. scapularis* [53], *I. ricinus* [54], *Haemaphysalis longicornis* [55] and *R. microplus* [56], and have not been described in other hard tick tissues. The role of SG metalloproteinases in tick feeding is thought to be linked to anti-fibrinogen, anti-febrin and anti-hemostatic activities [53]. The hypothesis is that tick salivary metalloproteases, together with other salivary anti-hemostatic proteins, may favor pathogen dissemination through vertebrate host tissues after transmission by ticks [57]. These findings may explain the observed up- and down-regulation of metalloproteases in response to *B. henselae* infection, by increasing bacterial dissemination after tick transmission for up-regulated genes, and by limiting this process as a host response to infection for down-regulated genes. The balance between these two processes may be essential for both bacterial and tick survival.

### Conclusion

Results from this study show that the *B. henselae/I ricinus* couple represents an excellent model for the study of molecular interactions between ticks and transmitted bacteria. Comparison of transcriptomes under controlled conditions between infected and non-infected ticks validated that differential transcript expression is due to the presence of the bacteria. Our data on differential expression of tick genes during bacterial infection represents the first comprehensive analysis of the molecular strategies employed by both ticks and bacteria to ensure their survival and development. However, because artificial membrane feeding systems were used, any natural vertebrate host responses were absent, and therefore their impact on the tick SG transcriptome could not be assessed.

Further detailed analysis of proteins encoded by genes identified in this study will contribute to a better understanding of the molecular dialogue between the two partners, an essential step in order to envisage TBP transmission blocking strategies. In addition, functional studies showed that silencing *IrSPI* expression reduced bacterial development as well as tick feeding, indicating that IrSPI may represent a very interesting candidate for vaccines against ticks and bacterial transmission. Further researches are now needed in order to evaluate the impact of silencing the corresponding gene in other infection models including pathogens belonging to the Anaplasma, Borrelia or Rickettsia genus. Indeed, highly effective anti-tick vaccines should reduce both tick burden and vector competence. The deployment of vaccines designed to reduce transmission of tick-borne pathogens by *I. ricinus* would represent a major improvement over current control measures as regards to environmental conservation and occupational exposure to tick-borne pathogens.

## Supporting Information

Table S1 **Differentially expressed isotigs between** ***Ixodes ricinus*** **salivary glands infected with** ***Bartonella henselae*** **and non-infected ones.** Both up-regulated and down-regulated isotigs following bacterial infection are presented in separate tabs with their corresponding best BlastX match to NR protein, annotation with GO terms, and allocation to various biological pathways using the KEGG server. Bir: *B. henselae* infected sample; Nir: non-infected sample; RA: relative abundance; FC: fold change; NA: not applicate.
(XLS)
